# CXCR4-targeted near-infrared imaging allows detection of orthotopic and metastatic human osteosarcoma in a mouse model

**DOI:** 10.1038/srep15244

**Published:** 2015-10-16

**Authors:** Guofeng Guan, Yao Lu, Xiaodong Zhu, Lijuan Liu, Jie Chen, Qiong Ma, Yinglong Zhang, Yanhua Wen, Lianjia Yang, Tao Liu, Wei Wang, Henry Ran, Xiuchun Qiu, Shi Ke, Yong Zhou

**Affiliations:** 1 Orthopedic Oncology Institute, Tangdu Hospital, Fourth Military Medical University, Xi’an, Shaanxi 710038, China; 2 Department of Microsurgery, Affiliated Hospital of Binzhou Medical University, Binzhou, Shandong 256603, China; 3 Department of Health Management, Affiliated Hospital of Binzhou Medical University, Binzhou, Shandong 256603, China; 4 Department of Radiology, Division of Molecular Imaging, Baylor College of Medicine, Houston, Texas 77030, USA; 5 Division of Epidemiology, Human Genetics and Environmental Sciences, The University of Texas Health Science Center at Houston, School of Public Health, Houston, Texas 77030, USA

## Abstract

CXCR4 is expressed at primary and metastatic sites of osteosarcoma. We developed a novel CXCR4-targeted near-infrared (NIR) fluorescent imaging agent (referred to as CXCR4-IR-783). The binding to representative osteosarcoma cells (F5M2 and F4 for high- and low- CXCR4 expression) was examined. CXCR4-IR-783 fluorescence was also examined in a mouse xenograft model of human osteosarcoma using NIR fluorescence microscopy and a Kodak *in-vivo* multispectral system. Pulmonary metastases in mice bearing osteosarcoma xenografts were detected by micro CT, ^18^F-PET scan and NIR imaging scan. Briefly, the binding of CXCR4-IR-783 was significantly higher in F5M2 than in F4 cells. Intense NIR fluorescence signals were detected in osteosarcoma xenografts, with signal/background ratio at 4.87 in mice bearing the F5M2 cell. At 4 weeks after F5M2 cell inoculation, metastatic lesions in the lungs were detectable using CXCR4-IR-783 and micro-CT scan, but not with ^18^F-FDG PET scan. In conclusion, CXCR4-IR-783 is a promising tool for detection of high CXCR4-expressing osteosarcoma, and particularly for its metastatic lesions.

Osteosarcoma is a common primary malignancy of the bone, and has a high rate of metastasis and recurrence^1^. With standard chemotherapeutic regimens, the 5-year survival rate is 60%–70% in patients with localized osteosarcoma^2^, but only 20% in patients with metastases^3^. Early diagnosis and treatment confer significant survival benefit^4^. Computed tomography (CT) and magnetic resonance imaging (MRI) are useful in detecting the lesions, but only when the lesions reach certain volume (typically one centimeter and >109 cells^5^,^6^).

Recent progresses in molecular imaging have expanded the capabilities of anatomical imaging methods^6^. CXCR4 is a receptor for the chemokine CXCL12 and is expressed at both primary and metastatic sites and it promotes metastasis, angiogenesis and growth of osteosarcoma cells^7^,^8^. A number of small molecules, peptides, and antibodies against CXCR4 have been developed for use in molecular imaging in the past few years^9^,^10^,^11^,^12^, but none has been used for osteosarcoma.

Near-infrared (NIR) fluorescence imaging has several advantages for cancer detection, including real-time display, high sensitivity, high spatial resolution and detailed molecular profiling^13^,^14^. Improvements in instrumentation, such as NIR intraoperative reflectance imaging, high-spatial resolution endomicroscopy and fluorescence tomography, have led to further improvement of NIR imaging^15^,^31^. NIR imaging agents such as indocyanine green have already been used for human breast cancer imaging and other clinical applications^16^,^17^. Meincke *et al.*^18^ have shown sensitive and selective binding of NIR fluorescent dye for CXCR4 to cancer cells in mouse xenograft models of human breast cancer and glioma.

In the current study, we synthesized a peptide that target to CXCR4 and labeled with IR-783 dye and developed a novel CXCR4-targeted NIR fluorescent imaging agent (CXCR4-IR-783). Then, we examined the selectivity and sensitivity of this agent using both cultured osteosarcoma cells and a mouse xenograft model of human osteosarcoma.

## Results

### Osteosarcoma cells overexpressing CXCR4 exhibit a preferential time- and dose-dependent uptake of CXCR-IR-783

**Figure 1:**
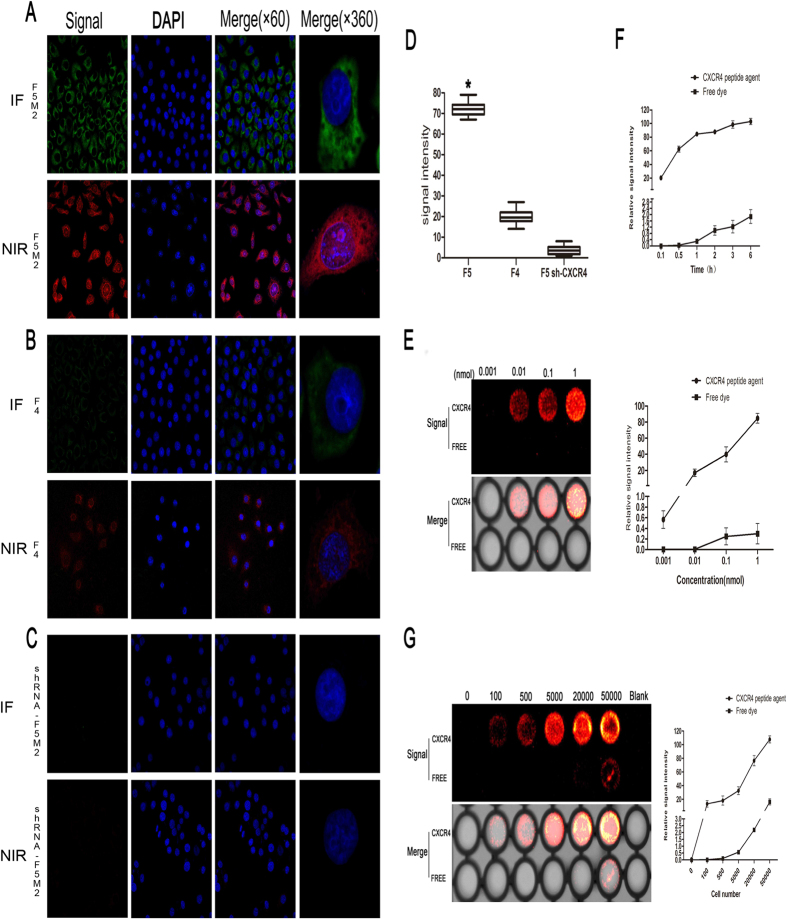
CXCR4-IR-783 is preferentially taken up by osteosarcoma cells overexpressing CXCR4 (A–F). F5M2 cells were transfected with shRNA against *CXCR4* as described in Methods. CXCR4 expression in F5M2 cells, F4 cells, and transfected F5M2 cells (shRNA-F5M2) was examined by immunofluorescent (IF) microscopy using anti-CXCR4 antibodies (green, (**A–C**)) upper leftmost panels). F5M2 cells, F4 cells, and transfected F5M2 cells were also treated with 5 nM CXCR4-IR-783 ((**A–C**) lower leftmost panels). Near-infrared (NIR) fluorescence was then examined as detailed in Methods. The nuclei were stained blue by DAPI (second left panels in (**A**–**C**). Merged images of anti-CXCR4 antibody staining or CXCR4-IR-783 fluorescence and DAPI staining are shown in the second right panels in (**A**–**C**). Magnification, 60 x, left three panels in (**A**–**C**). Merged images of single cells are shown in the rightmost panels in (**A**–**C**) at a magnification of 360 x. Mean NIR fluorescence intensity of F5M2 cells, F4 cells, and transfected F5M2 cells is shown in (**D**). Error bars represent SD of at least three independent experiments. *p < 0.0001 versus the other two groups, n = 10. Totally 1 × 10^4^ F5M2 cells were incubated with 10^−3^ to 1 nM CXCR4-IR-783 or free IR-783 for 1 h (**E**) or with 1 nM CXCR4-IR-783 or free IR-783 for up to 6 h at 37 °C (**F**). Moreover, 1 × 10^2^ to 5 × 10^5^ F5M2 cells were incubated with 1 nM CXCR4-IR-783 or free IR-783 for 1 h at 37 °C (**G**). NIR fluorescence was then visualized as described in Methods. Representative images are shown of at least three independent experiments in the left panels of (**E**,**G**). The data shown in (**F**) and the right panels of (**E**,**G**) are shown as mean ± SD of at least three independent experiments.

Real-time qRT-PCR and *Western blotting assays* showed significantly higher CXCR4 expression at both the mRNA and protein levels in F5M2 cells (with high propensity for pulmonary metastasis) than in F4 cells (with low propensity for pulmonary metastasis) (Supplementary Fig. S1A and S1B). Confocal immunofluorescent (IF) microscopy confirmed higher CXCR4 expression in F5M2 cells than in F4 cells (Figs 1A,B, upper panels). A polypeptide composed of 10 amino acids targeting CXCR4 was synthesized (Supplementary Fig. S2A) and then labeled with IR-783 dye to generate CXCR4-targeted NIR fluorescent imaging agent CXCR4-IR-783 (Supplementary Fig. S2B). Specificity of the NIR fluorescent imaging agent was confirmed by downregulation of CXCR4 expression with a shRNA against CXCR4 (Supplementary Fig. S1A and S1B; Fig. 1C, upper panel). Upon exposure to CXCR4-IR-783 (5 nM), the NIR fluorescent signal was significantly higher in F5M2 than in F4 cells (P < 0.05, Fig. 1D).

The NIR fluorescence intensity in F5M2 cells was increased by CXCR4-IR-783 (10^−3^ to 1 nM), but not by free IR-783, in a concentration-dependent manner (Fig. 1E): the signal was detectable at 10^−2^ nM and reached 102.2 ± 4.55 at 6 hr after exposure to 1 nM CXCR4-IR-783 (Fig. 1F). The signal also depended on the cell density within a range from 1 × 10^2^ to 5 × 10^5^ F5M2 cells (Fig. 1G).

### Mouse human osteosarcoma xenografts show preferential uptake and retention of CXCR4-IR-783

**Figure 2:**
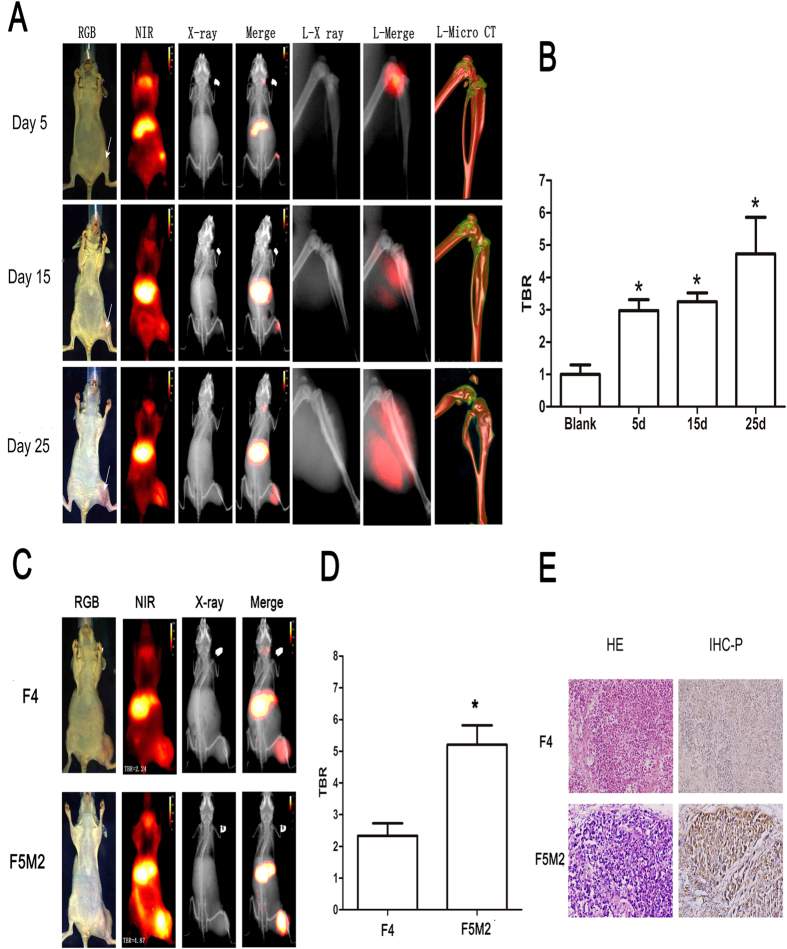
Mouse human osteosarcoma xenografts show preferential uptake and retention of CXCR4-IR-783. (**A**) Imaging studies were carried out 72 hours after injection of CXCR4-IR-783 at 5, 15 and 25 days post inoculation of F5M2 cells. The arrow in the leftmost RGB images indicates tumor location. Temporal changes of tumor sites are shown in the RGB images, the NIR images (red) of the whole body (NIR) or the tumor bearing leg (L-NIR), the X-ray images of the whole body (X-ray) or the tumor bearing leg (L-X ray) as well as CT skeleton images of the tumor bearing leg (L-Micro-CT). Merge or L-merge images of the NIR and X-ray images of the whole body or the tumor-bearing legs are shown by minimizing the extra-tumor signal of NIR. (**B**) The tumor-to-background ratios (TBR) at the tumor site in three separate time points show time-dependent increase of CXCR4-IR-783 uptake and retention by the tumor xenograft. N = 10, *p < 0.05 versus the blank control. (**C**) Comparison of NIR images of mice bearing F4 or F5M2 osteosarcoma xenografts 72 hours post injection of CXCR4-IR-783 at Day 25 post inoculation of the tumor cells. F4: mouse bearing F4 osteosarcoma xenograft; F5M2: mouse bearing F5M2 osteosarcoma xenograft. (**D**) The tumor-to-background ratios at the tumor site of mice bearing F4 and F5M2 osteosarcoma xenografts. *p < 0.05 versus F4. (**E**) H&E staining (left panel) and immunohistochemistry for CXCR4 (right panel) of the corresponding tumor tissue or normal tissues. Representative images are shown in (**A**,**C**) and compared at the same intensity scale.

Intense NIR fluorescence signal at the site of inoculation was detectable in mice on 5–25 days post inoculation with F5M2 cells (Fig. 2A), and the tumor-to-background ratio (TBR) remained stable throughout the 25-day experiments (Fig. 2B).The NIR fluorescence signal intensity was significantly higher in mice inoculated with F5M2 than with F4 cells (Fig. 2C, first and last row). The TBR was 4.87 vs. 2.24 for F5M2 and F4 xenografts, respectively (Fig. 2D). Pathological examination confirmed the presence of tumor in the bone (Fig. 2E). Immunohistochemistry (IHC) of tumor xenograft revealed higher CXCR4 expression in F5M2 xenografts than F4 xenografts (Fig. 2E). We also demonstrated distribution of the peptide agent in the whole body, including the liver and submandibular gland.

### CXCR4-IR-783 allows detection of lung metastasis

**Figure 3:**
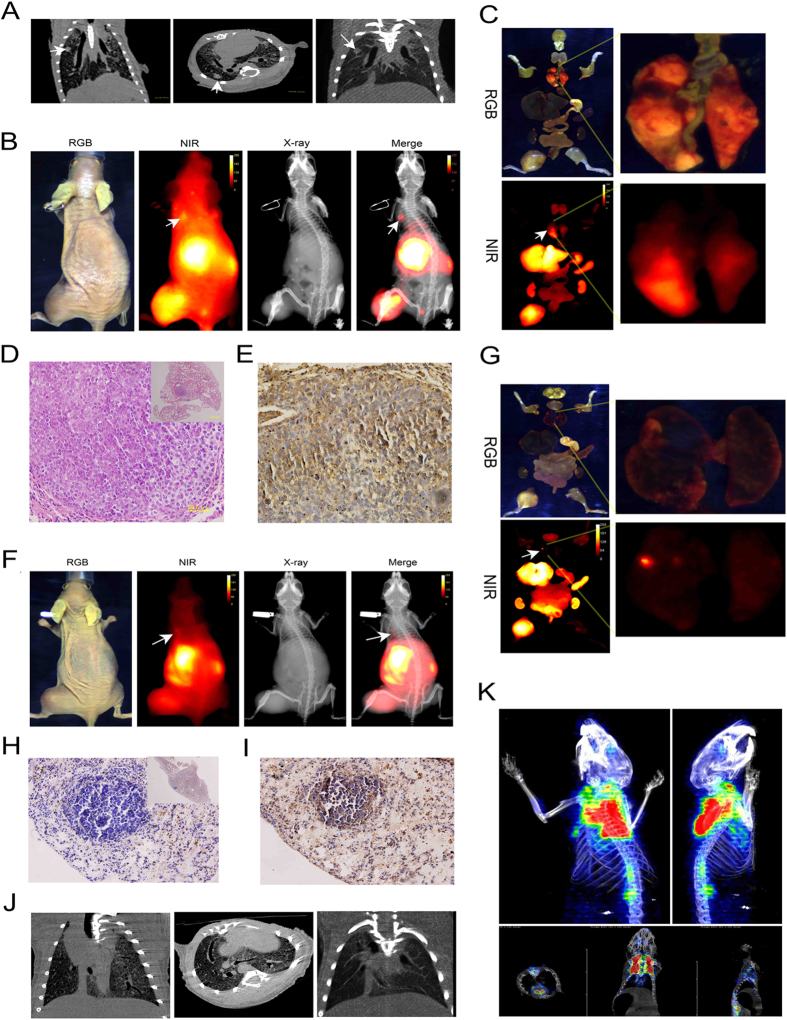
CXCR4-IR-783 allows detection of lung metastasis of osteosarcoma. (**A**) Micro -CT scan shows the presence of metastatic lesions (arrows) in the lungs of mice bearing F5M2 xenografts 6 weeks after inoculation. (**B**) The metastasis in the lungs is visualized by injection of CXCR4–IR-783 6 weeks after inoculation of F5M2 cells. The NIR image shows the presence of an apparent signal (arrow) in the lung, which is confirmed by the merged image of NIR and X-ray images. (**C**) RGB (upper panel) and NIR (lower panel) signal of all organs (left panel) and dissected lung tissue (right panel) of mouse bearing F5M2 cells 6 weeks after inoculation. (**D**) H&E staining reveals the presence of osteosarcoma cells in the lung tissues (400 × and 40 × (inset)). (**E**) Immunohistochemistry shows apparent CXCR4 expression in the metastatic osteosarcoma tissue (400 × ). (**F**) NIR fluorescence signal (arrow) is detected in the lungs of mouse bearing F5M2 xenografts 4 weeks post tumor implantation. (**G**) The RGB (upper panel) or NIR (lower panel) images of all *ex-vivo* organs (left panel) and lung tissue (right panel). The presence of osteosarcoma metastasis in the lung tissues is confirmed by histopathologic evaluation with H&E staining (H, 400 × and 40 × (inset)) and by immunohistochemistry for CXCR4 (I, 400 × ). The metastatic lesions in the lungs could be detected using Micro-CT scan (**J**) and ^18^F-FDT PET scan (**K**) of the chest failed to reveal the high glucose uptake of the nodules in the lungs.

Our previous study^19^ showed that the metastatic nodules in the lungs became detectable six weeks after inoculation of F5M2 cells. In the current study, micro-CT scan revealed nodules in the lungs at 6 weeks after inoculation with F5M2 cells (Fig. 3A). NIR imaging revealed fluorescent signal in the lungs of mice bearing F5M2 xenografts, at sites suggested by CT imaging (Fig. 3B). *Ex vivo* imaging confirmed the NIR signals in the submandibular gland, lung metastatic tumor, liver, kidney and primary tumor (Fig. 3C). The presence of osteosarcoma cells in the lung tissues was confirmed by histopathologic evaluation with H&E staining (Fig. 3D). CXCR4 expression in the metastatic osteosarcoma tissue was demonstrated by IHC (Fig. 3E). When molecular imaging showed negative results (Supplementary Fig. S3A and S3B), histopathologic examination also confirmed the absence of lung nodules (Supplementary Fig. S3C). In IHC analysis, CXCR4 expression was not detectable in healthy lungs (Supplementary Fig. S3D). NIR fluorescence signal appeared in the lungs of mice bearing F5M2 xenografts as early as four weeks post tumor implantation (Fig. 3F) with < 1 mm (as small as 200 μm) lung metastatic nodules (Fig. 3G). Micro-metastasis in the lungs was confirmed by histopathologic evaluation with H&E staining (Fig. 3H) and by IHC for CXCR4 (Fig. 3I). Micro-CT scan also detected micro-metastasis in the lungs (Fig. 3J) but ^18^F-FDT PET scan of the chest failed to reveal any change in glucose uptake (Fig. 3K).

### CXCR4-IR-783 exhibits time-dependent clearance from normal mouse organs

**Figure 4:**
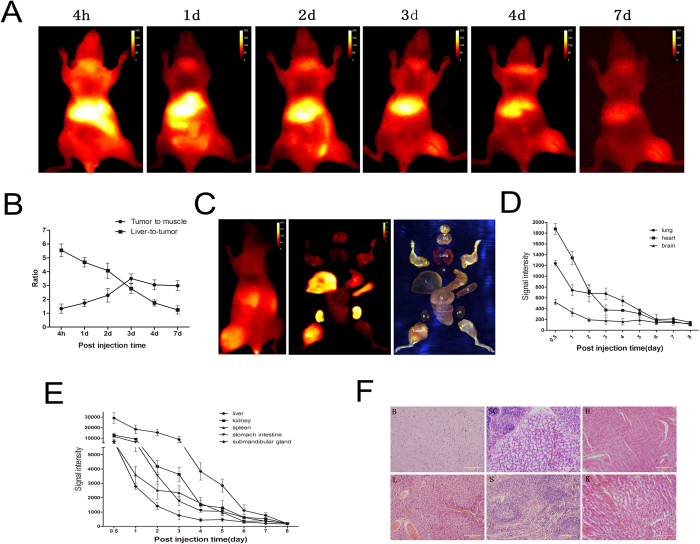
CXCR4-IR-783 exhibits time-dependent clearance from normal mouse organs. (**A**) The temporal distribution of CXCR4-IF-783 in mice bearing F5M2 osteosarcoma xenografts three weeks after tumor implantation by contiguous NIR imaging. Animals are shown in the ventral view. (**B**) The tumor-to-muscle (pellets) and liver-to-tumor ratio (squares) ratios following injection of CXCR4-IF-783. Data shown are mean ± SD. N = 10. (**C**) The NIR image *in vivo* (left) and *ex vivo* (middle), and the RGB image show the corresponding dissected organs (right). Contiguous NIR imaging demonstrates a steady decline in the mean NIR fluorescence intensity in the lungs, heart and brain (**D**) and the liver, spleen and other organs as well as the tumor xenograft from the day of injection of CXCR4-IR-783 to day 8 post injection (**E**). (**F**) H&E staining confirms the tissue type in the organs. B, brain, SG, submandibular gland, H, heart, L, liver, S, spleen and K, kidney (K) (400 × ).

The tumor and muscle ratio of CXCR4-IR-783 increased from day 1 and peaked at day 3 post injection of CXCR4-IR-783 in mice bearing F5M2 xenograft (Fig. 4A,B). The liver-to-tumor ratio of CXCR4-IR-783 reached a peak value (5.5 ± 0.7) at 4 hours and declined to 1.1 ± 0.2 at day 7 post injection (Fig. 4B). Contiguous NIR imaging showed a steady decline in NIR fluorescence intensity in the lungs, heart and brain (Fig. 4C,D) and the liver, spleen and other organs (Fig. 4E). Histological examination failed to reveal the presence of metastatic osteosarcoma in these organs, including the submandibular gland and liver, (Fig. 4F).

## Discussion

Lack of effective imaging modalities for detection of clinically non-detectable micro-metastases of osteosarcoma hampers its management. NIR fluorescence cancer imaging has demonstrated excellent sensitivity and high spatial resolution with real-time display^13^,^14^. In the current study, we developed a novel CXCR4 targeted NIR fluorescent imaging agent (CXCR4-IR-783). This agent exhibited superior selectivity in detection of CXCR4 expression in osteosarcoma cells both *in vitro* and *in vivo*. More importantly, NIR fluorescence imaging using CXCR4-IR-783 detected the pulmonary metastases of osteosarcoma that were not detected by ^18^F-FDG PET. NIR fluorescence cancer imaging using a human recombinant SDF-1 conjugated with IRDye800CW has been attempted for breast cancer in a mouse xenograft model^18^, but there has been no prior study on the use of CXCR4-IR-783 for imaging osteosarcoma and its metastases. Our study provides direct evidence that NIR fluorescence imaging using CXCR4-IR-783 visualizes osteosarcoma and its metastases.

NIR imaging uses near-infrared light (wavelength: 650–900 nm), and thus has deeper tissue penetration due to less absorption by hemoglobin and water, and low background due to less autofluorescence from surrounding tissues^13^,^20^,^21^. Moreover, NIR dyes such as indocyanine green have low and negligible toxicity and are widely used in clinical applications, including cancer detection/characterization, lymphatic imaging and surgical/endoscopic guidance^13^,^14^,^20^,^22^. CXCR4 is implicated in tumor progression, including angiogenesis and metastasis^23^,^24^. Increasing evidence indicates that CXCR4 is expressed in various stages of tumor and its expression in the primary lesions correlates with metastasis and poor survival of patients with breast cancer, cervical adenocarcinoma and other cancer types^12^,^24^,^25^. CXCR4 is also expressed in primary and metastatic lesions of human osteosarcoma and associated with metastasis and poor prognosis of osteosarcoma^7^,^26^.

Molecular imaging is highly dependent on a specific molecular target^14^,^27^. A successful imaging agent should exhibit consistent binding affinity for its molecular target regardless of the mutation status at the protein level^28^. In the current study, the peptide agents, CXCR4-IR-783, were screened, minimized and modified to ensure the final product binding to the conservative region of CXCR4 receptor. Compared to free IR-783, CXCR4-IR-783 was associated with significantly higher NIR fluorescence intensity in F5M2 cells. Meanwhile, we demonstrated a higher uptake of CXCR4-IR-783 by osteosarcoma cells overexpressing CXCR4 (F5M2) than osteosarcoma cells expressing low level of CXCR4 (F4). Our peptide imaging yielded similar results both *in vitro* and *in vivo*. The two cell lines were derived from the same parental osteosarcoma cell line SOSP-9607^19^, suggesting that difference in CXCR4 expression is responsible for binding difference. Furthermore, a *CXCR4* shRNA markedly attenuated CXCR4-IR-783 NIR fluorescence intensity in F5M2 cells, adding further support to the binding specificity of CXCR4-IR-783.

Upon presentation, approximately 80% of osteosarcoma patients have metastatic diseases, and many patients also have undetectable pulmonary micro-metastases^29^. Yet, only a very small portion of these patients are diagnosed with the current methods^29^,^30^. In our experiments, the only clinically available molecular imaging modality, ^18^F-FDG PET, failed to show increased glucose uptake in the metastatic lesions. ^18^F-FDG PET imaging relies on glucose level to differentiate disease region but the limitation of spatial resolution of PET detector and low glucose metabolic status make it impossible in this study. Micro-CT provides approximate robust anatomical information and evidence for highly probable metastatic lesions in the lungs. However, it cannot detect the disease components at the molecular level. Determination of the disease components at the molecular levels could support CT findings since these lesions tend to appear as healthy tissue in X-ray. In the current study, NIR fluorescence imaging using CXCR4-IR-783 detected micro-metastasis as small as 200 μm in diameter, indicating that CXCR4-targeted imaging could improve detection of CXCR4-positive osteosarcoma metastasis.

CXCR4 is widely expressed in monocytes and macrophages, and normal tissues such as the colon, kidney, and liver^23^. Our *in vivo* successive imaging showed that the tumor to muscle ratio peaked at 3 days after injection, and exceeded the liver-to-tumor ratio. *Ex vivo* distribution analysis revealed that, in addition to the tumor, the signal concentrated in the liver, kidney, spleen, stomach, intestine, and submandibular gland upon initial injection of CXCR4-IR-783, but the signal intensity decreased over time, suggesting that, within proper window, this probe could be used to detect osteosarcoma with relative high signal-to-noise ratio. Although histological examination failed to reveal the presence of metastatic osteosarcoma in the submandibular and liver, the presence of NIR signal in these organs is too high and requires further investigation. So CXCR4-IR-783 is not optimal. Further modification of the probe is currently carried out in our laboratory.

In conclusion, we developed a novel CXCR4-targeted NIR fluorescent imaging agent (CXCR4-IR-783). This probe could detect both primary and micro-metastatic lung lesions of CXCR4-overexpressing osteosarcoma. NIR fluorescence imaging with this agent, combining with other imaging modalities (such as X-ray) which can provide anatomical information, will enhance the detection of osteosarcoma.

## Materials and Methods

### Ethics statement

All the animal operations were approved by the Ethics Review Committee of the Fourth Military Medical University, Xi’an, Shaanxi, China. The methods were carried out in accordance with the approved guidelines.

### Cells and lentiviral infections

F5M2, a human osteosarcoma cell line with high propensity for pulmonary metastasis, and F4, a human osteosarcoma cell line with low propensity for pulmonary metastasis, were previously described^19^ and grown in RPMI 1640 (HyClone, Thermo Scientific, Waltham, Massachusetts) supplemented with 10% fetal bovine serum (FBS), penicillin (100 units/mL), streptomycin (100 μg/mL), and glutamine (2 mM).

For generation of stable *CXCR4*-knockdown cells, lentivirus vectors expressing short-hairpin RNA (shRNA; sequence, 5′-TCCTGTCCTGCTATTGCATTA-3′) targeting *CXCR4* (Hanbio Biotechnology, Shanghai, China) were transfected into 293 T cells using Lipofectamine 2000 (Invitrogen, Carlsbad, California) and lentiviral packaging mix according to the manufacturer’s protocol. F5M2 cells were then transfected with the lentiviruses at a multiplicity of infection (MOI) of 100. Stable *CXCR4*-knockdown cells were selected with puromycin (3 mg/mL) (Hanbio Biotechnology) and confirmed real-time quantitative reverse transcription polymerase chain reaction (qRT-PCR) and Western blotting assays as detailed elsewhere in the text.

### Immunofluorescence confocal microscopy

Cells were plated onto Millicell EZ SLIDE 8-well glass (Millipore, Darmstadt, Germany) for 12 h and then fixed with 4% paraformaldehyde followed by permeabilization with phosphate buffered saline (PBS) containing 0.1% Triton X-100 (PBST). After blocking with normal goat serum, the cells were incubated with anti-CXCR4 antibodies (R&D Systems) at 4 °C overnight. After three washes with 0.1% PBST, the cells were incubated with secondary FITC-conjugated sheep anti-mouse IgG (Sigma) and mounted with DAPI (Sigma) after wash with PBST. The cellular localization and expression of CXCR4 were examined under a confocal microscope (Olympus, Tokyo, Japan).

### NIR fluorescence microscopy

The peptide agents were screened, minimized and modified to ensure the final product binding to the conservative region of CXCR4 receptor. Such approach will maintain the binding capability even after the cancer cells mutation during their progression. A detail design, modification, stability, structure change the binding affinity and pathway function for different structure will be summarized and published in a separate chemistry journal. Briefly, a peptide agent that target to CXCR4 was synthesized at Department of Radiology, Baylor College of Medicine, Houston, TX using Fmoc-based solid-phase peptide synthesizer and labeled with IR-783 dye. The conjugate was purified by reverse-phase high-performance liquid chromatography (HPLC) and was certificated by mass spectrometry, analytic HPLC, and fluorescent spectrophotometry. Exponentially growing F5M2 and F4 cells were seeded at a density of 2 × 10^4^ cells/well in 4-well glass slides (Millipore) in RPMI-1640 containing 10% FBS. After an overnight growth, the cells were treated with IR-783 or CXCR4-IR-783 over a 1000-fold range from 10^−3^ to 1 nM or the indicated periods of time as detailed elsewhere in the text. Then, the cells were washed thrice with PBS. After fixation with 4% formaldehyde, the cell nuclei of the cells were stained by DAPI. Images were captured using a Leica TCS confocal microscope (Leica TCS SP5, Germany) equipped with an excitation light source and emission filters to detect and separate signals from near infrared agent (IR-783-CXCR4 and IR-783) and DAPI. Images were scanned and captured at 60× and 360× magnifications and mean fluorescence intensity was calculated.

### Animal xenograft studies

Four-week-old female BALB/c nude (nu/nu) mice (the Animal Center, the Fourth Military Medical University, Xian, Shaanxi, China) were housed under pathogen-free conditions at 26–28 °C with 50–65% humidity. Totally 5 × 10^5^ F5M2 or F4 cells in 50 μL PBS were injected into the right hind tibia as previously descripted^19^. Tumors were allowed to grow six weeks to allow metastasis. No mice died before six weeks.

### Animal imaging studies

Mice bearing tumor xenografts were injected intravenously with IR-783-CXCR4 or IR-783 200 μl a mouse. Imaging was performed 4 h to 9 days after the injection using Kodak *In-Vivo* Multispectral System FX (Carestream Health Molecular Imaging, New Haven, Connecticut) and the Carestream Molecular Imaging (MI) Software while the mice were immobilized by inhalational isoflurane (Jiu Pai Laboratories, China) and kept warm at 37 °C. Excitation and emission wavelengths were fixed at 760 and 830 nm, respectively. In addition, *ex vivo* imaging was performed after the removal of organs or tumors following sacrifice of the animals by cervical dislocation. The images and data were analyzed using the Carestream Molecular Imaging (MI) Software and the open-source software Image J (http://rsbweb.nih.gov/ij/) to distinguish autofluorescence from the normal tissues and fluorescence from the tumors. Fluorescence intensity and tumor-to-background ratio, which was defined as the fluorescence intensity ratio between tumor and background autofluorescence, were automatically calculated by the software. Mice were also imaged on a Siemens Inveon Micro CT (Siemens, Munich, Germany). Three dimension CT images or movies were reconstructed by Inveon Acquisition Workplace (Siemens, Munich, Germany). For detection of metastatic nodules, Inveon PET (Siemens Medical Solutions, Malvern, Pennsylvania) was used to detect ^18^F-FDG signal in tumor in the lungs.

### Immunohistochemistry

Tumor tissue specimens were fixed in 4% paraformaldehyde and embedded in paraffin and then sectioned (4 μm in thickness). Immunohistochemistry was performed using the IHC-P method as instructed by the manufacturer (Abcam, Cambridge, Massachusetts). The tissue sections were incubated with monoclonal mouse anti-human CXCR4 antibodies (R&D Systems), and then with multi-use secondary antibody (Dako, Glostrup, Denmark). The tissue sections were visualized with the EnVisionTM Peroxidase/DAB Rabbit/Mouse detection kit (Gene Tech, Shanghai, China). In negative controls, PBS was used instead of primary antibodies. The immunostaining results were evaluated as previously described^7^.

### Statistical analysis

All data were presented as mean ± SD and analyzed using the SPSS 13.0 software (SPSS Inc., Chicago, Illinois). Regions of interest (ROIs) were quantified using the open-source software Image J (http://rsbweb.nih.gov/ij/). Fluorescence intensity of different tissues was defined as the mean fluorescence intensity of the ROI. Tumor-to-background ratios were calculated as previously described^28^. Significance of difference was analyzed with a two-sided Student’s *t* test and *p* < 0.05 were considered statistically significant.

## Additional Information

**How to cite this article**: Guan, G. *et al.* CXCR4-targeted near-infrared imaging allows detection of orthotopic and metastatic human osteosarcoma in a mouse model. *Sci. Rep.* **5**, 15244; doi: 10.1038/srep15244 (2015).

## Supplementary Material

Supplementary Information
