# Supplementary material for: CXCR4-targeted near-infrared imaging allows detection of orthotopic and metastatic human osteosarcoma in a mouse model
Source: Sci Rep. 2015 Oct 16;5:15244. doi: 10.1038/srep15244 (PMC4607947; doi:10.1038/srep15244)
Supplement: Supplementary Information [file srep15244-s1.doc]

**CXCR4-targeted near-infrared imaging allows detection of orthotopic and metastatic human osteosarcoma in a mouse model**

**Running title: CXCR4-targeted near-infrared imaging in osteosarcoma**

Guofeng Guan^a,e,1^, Yao Lu^a,1^, Xiaodong Zhu^e,1^, Lijuan Liu^b,1^, Jie Chen^a^, Qiong Ma^a^ , Yinglong Zhang^a^, Yanhua Wen^a^, Lianjia Yang^a^, Tao Liu^a^, Wei Wang^c^, Henry Ran^c^, Xiuchun Qiu^a^,*, Shi Ke^c,d^,*, Yong Zhou^a^,*

^a^Orthopedic Oncology Institute, Tangdu Hospital, Fourth Military Medical University, Xi'an, Shaanxi 710038, China

^b^Department of Health Management, Affiliated Hospital of Binzhou Medical University, Binzhou, Shandong 256603, China

^c^Department of Radiology, Division of Molecular Imaging, Baylor College of Medicine, Houston, Texas 77030, USA

^d^Division of Epidemiology, Human Genetics and Environmental Sciences, The University of Texas Health Science Center at Houston, School of Public Health, Houston, Texas 77030, USA

^e^Department of Microsurgery, Affiliated Hospital of Binzhou Medical University, Binzhou, Shandong 256603, China

^1^These authors contributed equally to this work.

*Corresponding author

**Correspondence:** Yong Zhou, Orthopedic Oncology Institute, Tangdu Hospital, Fourth Military Medical University, 569 Xinsi Road, Xi'an, Shaanxi 710038, China.

Tel.: +8613709215796; Fax:+86-029-8477-8356; E-mail: gukezy@fmmu.edu.cn.

Shi Ke, The University of Texas Health Science Center at Houston, School of Public Health, Houston, Texas 77030, USA

E-mail: [shike888@gmail.com](mailto:shik@bcm.edu)

Xiuchun Qiu, Orthopaedic Oncology Institute, Tangdu Hospital, Fourth Military Medical University, 569 Xinsi Road, Xi'an, Shaanxi, China.

E-mail: qiuxiuchun2009@live.cn

**Supplementary Methods**

*Real-time quantitative RT-PCR*

Total cellular RNA was isolated using the Trizol reagent (Invitrogen) as instructed by the manufacturer. First-strand cDNA was synthesized using the *AMV* First Strand cDNA Synthesis Kit (Sangon Biotech, Shanghai, China) and oligo (dT) following the manufacturer’s instruction. Real-time qRT-PCR was performed with the LightCycler480 software (Roche) with the ABI SybrGreen PCR Master Mix (Sangon Biotech). Relative gene expression was calculated with the Ct 2^-ΔΔCT^ method. The sequences of the primers used in the study were as follows: CXCR4, 5’-AATAAAATCTTCCTGCCCACC-3’ (sense) and 5’-CTGTACTTGTCCGTCATGCTTC-3’ (anti-sense); β-actin, 5’-TAGTTGCGTTACACCCTTTCTTG-3’ (sense) and 5’-TCACCTTCACCGTTCCAGTTT-3’ (antisense). β-Actin was used as an internal housekeeping gene. All experiments were performed at least three times independently in triplicate.

*Western blotting assays*

Cellular lysates were prepared using pre-chilled lysis buffer (1% Trition X-100, 50 mmol/L Tris-Hcl, pH 7.4, 1 mmol/L EDTA, 150 mmol/L NaCl, 2 mmol/L PMSF, and 1 mmol/L sodium orthovanadate). The lysates were clarified by centrifugation at 12000 rpm for 15 min and resolved by SDS denatured polyacrylamide gel electrophoresis. The immunoblotting procedure was performed as previously detailed (1). Mouse monoclonal anti-CXCR4 (Abcam, UK) and anti-β-actin antibodies (Sigma, St. Louis, MO) were used. CXCR4 expression was normalized against that of β-actin.

**Supplementary figure legends**

**Figure 1** **Expression of CXCR4 in osteosarcoma cells.** F5M2 cells were treated with short hairpin RNA (shRNA) against CXCR4 as described in Methods. The mRNA transcript levels of CXCR4 in F5M2, F4, and shRNA-treated F5M2 cells were determined by quantitative RT-PCR (A). CXCR4 expression was normalized against β-actin. Protein expression of CXCR4 in the above cells was determined by Western blotting assays and normalized against β-actin (B). Data shown in (A) are mean ± SD of at least three independent experiments performed in triplicate. ***p*<0.05. Immunoblots shown in (B) are representative of data of at least three independent experiments.

**Figure 2** (A) structure of CXCR4 peptide; (B) structure of CXCR4-IR-783.

**Figure 3** (A) The NIR fluorescence signal in the lungs of mice bearing F5M2 xenografts three weeks post tumor implantation. (B) The RGB and NIR signal of all organs (left panel) or dissected lung tissue (right panel). (C) H&E staining shows lack of any histological evidence for lung metastasis (400× and 40× (inset)). (D) Immunohistochemistry reveals no expression of CXCR4 in the lung tissue (400×).

**Supplementary Figure 1**


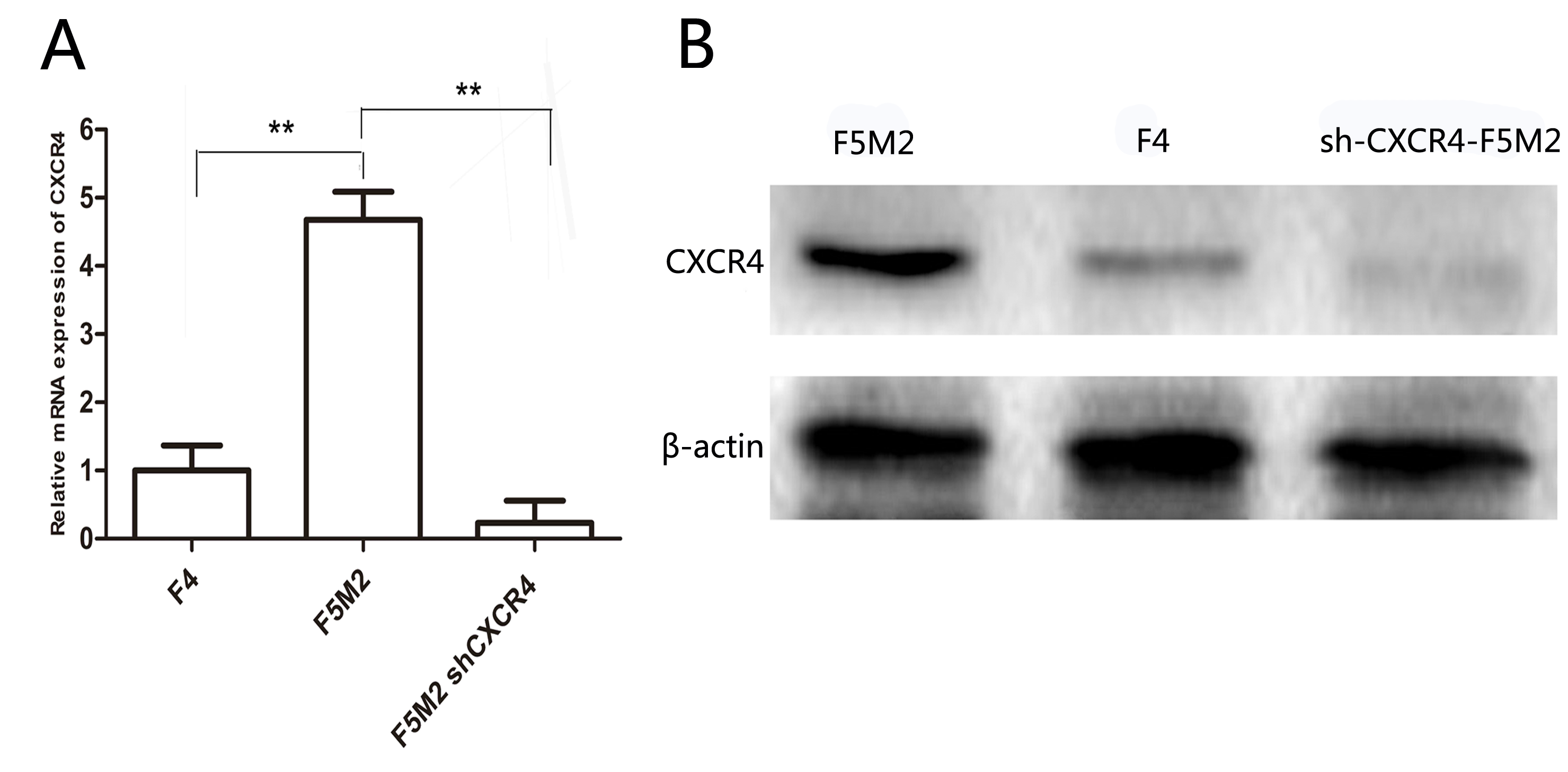


**Supplementary Figure 2**

**
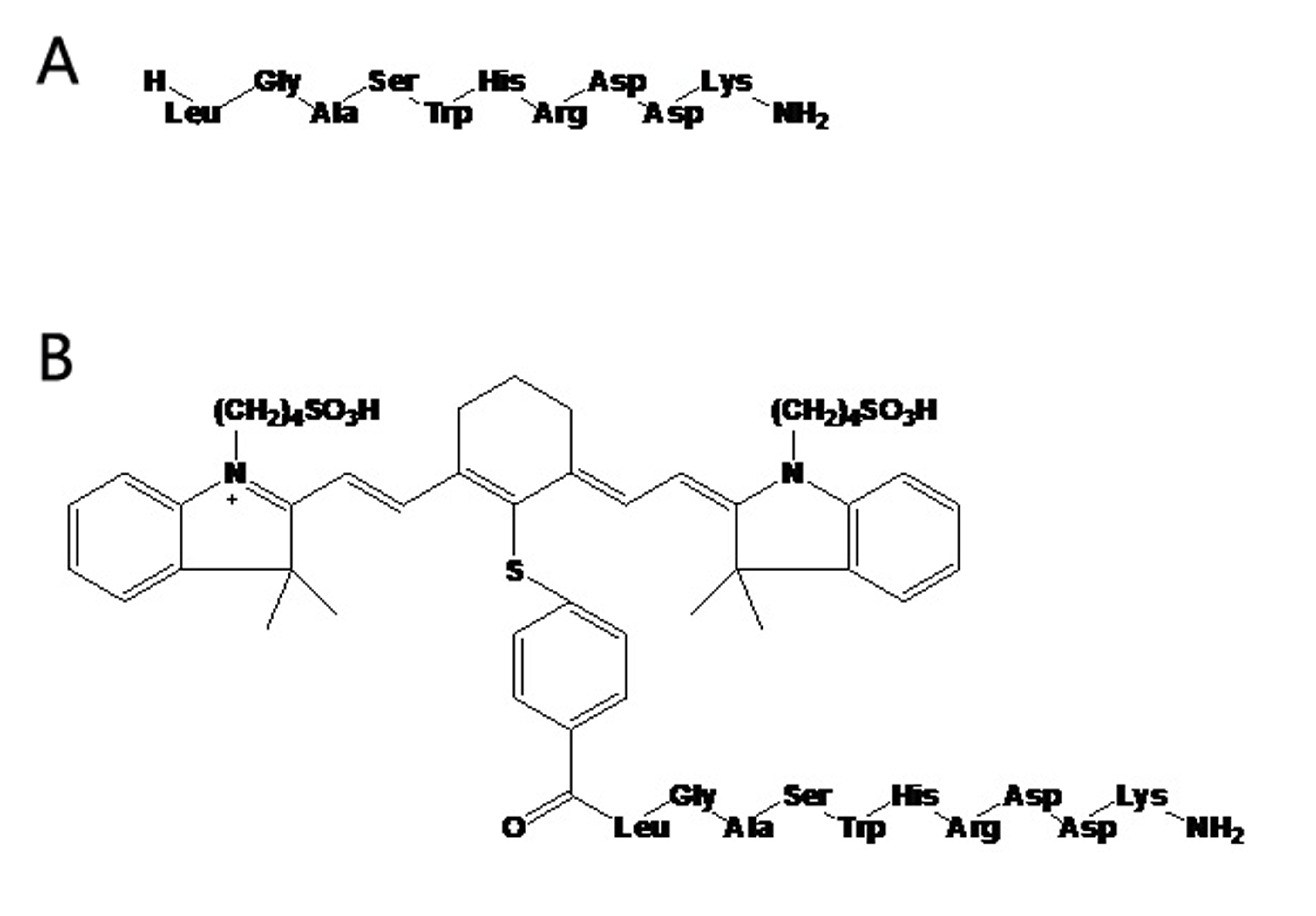
**

**Supplementary Figure 3**

**
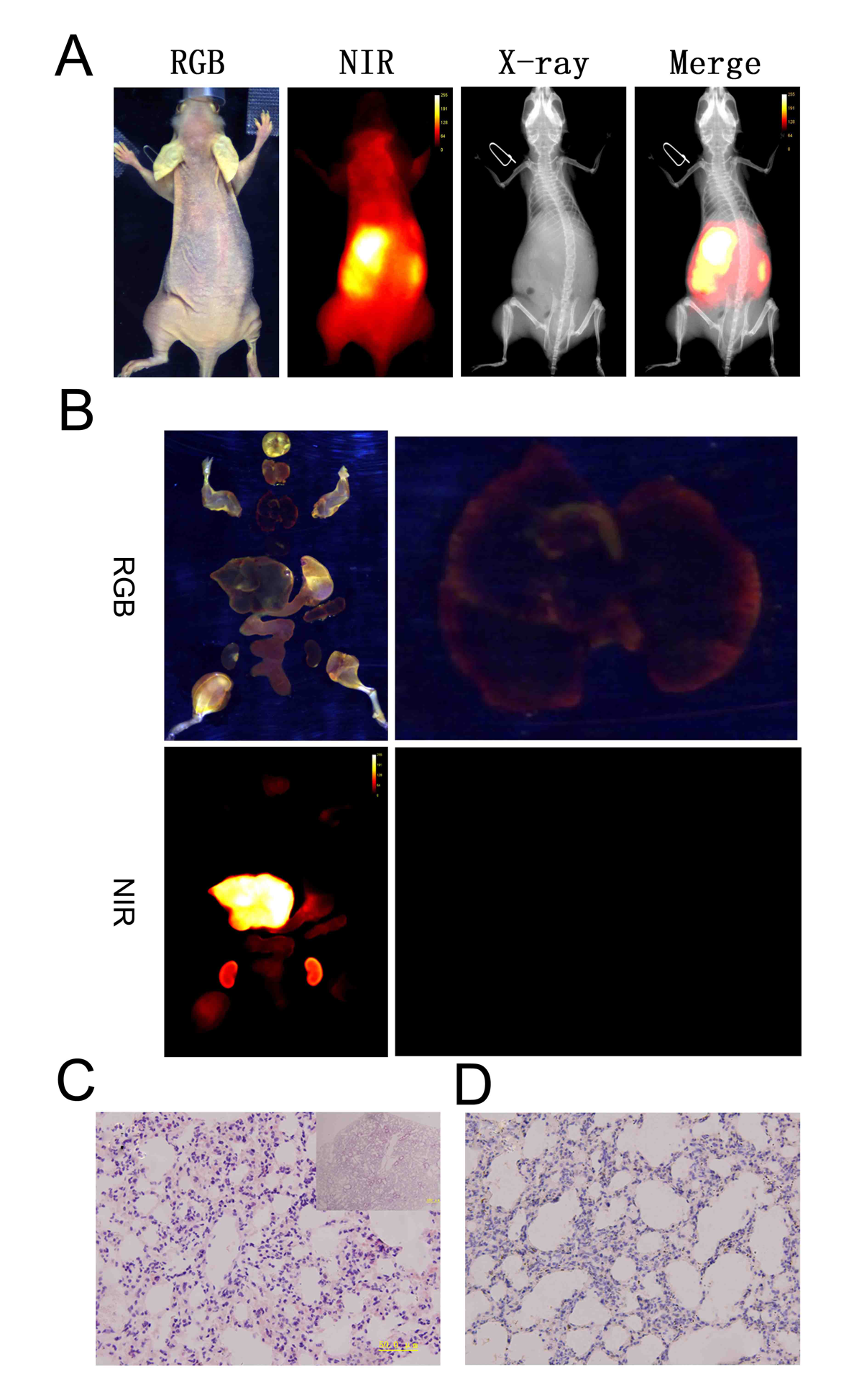
**
